# Investigation of Geographical Differences of Arabinoxylan in Wheat Grain and Gel Properties of Arabinoxylan/Starch Complexes and In Vitro Digestion

**DOI:** 10.3390/foods13244060

**Published:** 2024-12-16

**Authors:** Haixia Wu, Ting Zhou, Ruifeng Ying, Yuanlin Sun

**Affiliations:** 1 Department of life Science, Yuncheng University, Yuncheng 044000, China; sylwts@aliyun.com; 2 Department of Food Science and Technology, College of Light Industry and Food Engineering, Nanjing Forestry University, Nanjing 210037, China; zhoutingnjfu@163.com (T.Z.); yingruifeng@njfu.edu.cn (R.Y.)

**Keywords:** Arabinoxylan, wheat starch, gelatinization, retrogradation, digestion

## Abstract

With an increasing number of people pursuing a healthy diet, people have gradually realized the significance of adequate dietary fiber in their diets. In this experiment, wheat bran was collected from eight regions in China with different longitudes and latitudes, different altitudes, and average temperatures during the filling period to study the differences in the Arabinoxylan (AX) of wheat bran. The higher the altitude of the wheat production area was, the higher the AX content in the wheat bran was. Therefore, wheat bran from high-altitude production areas was selected for extracting AX. Different proportions of AX were added to wheat starch (WS) to explore the influence of different concentrations of AX on the gelatinization of WS, including the solubility, swelling capacity, rheological properties, and microstructure of the gelatinized products. Among these eight kinds of wheat, the content of total AX accounted for 11.90–15.79% of their dry weight, with the highest content being in wheat from Wuwei, Gansu. Among them, the content of water-soluble AX accounted for approximately 0.85% of their dry weight content. After adding different concentrations of 0.05–2% AX to the WS system, the gel network structure was changed. The starch hydrolysis rate of bread with 2% AX added was the lowest, of which the contents of rapidly digestible starch and slowly digestible starch were 40.02% and 36.61%, and resistant starch was as high as 25.31%. The addition of AX to starch-based foods is helpful for controlling postprandial blood sugar and insulin levels.

## 1. Introduction

Cereal foods constitute an important part of the human diet [1]. Wheat, as a major food crop, provides 19% of the calories and 20% of the proteins in the global human diet [2,3]. With the increasing pursuit of a healthy diet by more and more people, the importance of adequate dietary fiber in diets has gradually been recognized [4,5]. The main types of dietary fiber include resistant starch, cellulose, hemicellulose (including xylan, pectin, and β-glucan), and oligosaccharides [6].

Arabinoxylan (AX), as a typical dietary fiber in cereals [7], is a polysaccharide present in the cell walls of grains such as wheat, barley, and highland barley. It has various physiological activities, such as regulating blood sugar and blood lipids, promoting intestinal health, and inhibiting cholesterol absorption [8,9]. AX consists of a linear backbone of xylose units and attached arabinose units [6].The general structure of AX contains a linear (1→4)-β-D-xylopyranoside (Xylp) unit chain, with α-L-arabinofuranosyl units attached thereon via α-(1→2) and/or α-(1→3) glycosidic bonds. Therefore, there are mono- and/or di-substituted Xylp residues at the O-2 and O-3 positions.

Adding AX to wheat starch [10] not only can change the processing characteristics of starch-based foods [11,12] but also contributes to a healthy human diet [6,13]. For example, the addition of soluble AX in bread improves dough parameters and bread quality, increasing the water absorption rate and volume of bread and reducing the hardness of breadcrumbs. Its stickiness maintains the stability of air cells in the dough by influencing the gluten starch [14,15].

Previous studies have shown that the physicochemical properties of AX are related to its specific molecular structure [16]. Among them, the solubility of AX is related to the degree of branching and the relative molecular weight (M_w_) [17]; AX with a higher substitution rate is more soluble, which may be due to the tendency of the unsubstituted part of AX to aggregate through hydrogen bonds, resulting in a decrease in solubility [18]. When the branching levels are similar, the lower the M_w_ is, the lower the solubility will be [19]. Water-soluble AX can act as an indirect emulsion stabilizer by cross-linking surface-active component proteins [20,21]. Kaur et al. [22] found that the AX extracted by alkali has better emulsifying ability than the water-extracted AX (WEAX), which may be attributed to a larger molecular weight.

The rapid digestion of gelatinized starch granules in the human gastrointestinal tract will increase blood sugar levels related to oxidative stress and insulin resistance [23,24] and induce many chronic health problems such as obesity and type II diabetes [25,26]. Therefore, slowing down the digestion of starch by digestive enzymes in the gastrointestinal tract and stabilizing blood sugar levels are of great significance for preventing the occurrence of chronic diseases [27,28]. Previous studies have found that starch digestion is affected not only by its internal structure but also by the interactions between starch and non-starch compounds and between digestive enzymes and non-starch compounds [29,30]. Yan et al. [31] found that AX with a larger molecular weight and degree of branching can effectively reduce the water utilization rate of starch gelatinization and hinder the gelatinization process of wheat starch (WS). WEAX can increase the G′ and G″ of wheat dough and significantly improve gel strength. WEAX inhibits the recrystallization of amylose, thereby inhibiting the short-term retrogradation of amylose; however, high-molecular-weight WEAX mainly delays the recrystallization of amylopectin and has a more significant inhibitory effect on the long-term retrogradation of amylopectin [11,31,32,33].

In this experiment, wheat bran was collected from eight different regions in China with different longitudes and latitudes, different altitudes, and average temperatures during the filling period to study the differences in the AX content of wheat bran. We hoped to find wheat bran with a high content of AX and extract AX from this wheat bran. Then different proportions of AX would be added to WS to explore the effects of different concentrations of AX on the gelatinization of WS, including the solubility, swelling capacity, rheological properties, and microstructures of the gelatinized complexes.

## 2. Materials and Methods

### 2.1. Materials

The wheat used was all harvested in the autumn of 2022, and its basic information is shown in Table 1. Wheat starch was purchased from Xinxiang Liangrun Co., Ltd., Xinxiang, China. Glacial acetic acid, sodium hydroxide, sodium potassium tartrate, 3, 5-dinitrosalicylic acid, phenol, and anhydrous sodium sulfite were purchased from Sinopharm Chemical Reagent Co., Ltd., Shanghai, China. Porcine pancreatic enzymes (3800 U/mg), α-amylase (8 U/mg), and pepsin (30 U/mg) were purchased from Shanghai Yuanye Biotechnology Co., Ltd., Shanghai, China Iodine, potassium iodide (≥99%), and starch glucosidase (6 U/mg) were purchased from Shanghai Macklin Biochemical Technology Co., Ltd., Shanghai, China.

### 2.2. Extraction of AX and Determination of Its Basic Physicochemical Properties

AX was extracted from wheat bran aleurone layer by hot water–alkali extraction method [34].

The molecular weight of the extracted AX was analyzed by the 2414 Waters differential refractive index detector (RID) using Waters e2695 high-performance liquid chromatography (HPLC, Waters, Milford, CT, USA). The volume-exclusion HPLC column was Thermo Scientific Acclaim SEC-1000, and the calibration range of the column was from 1000 to 1,000,000 Da. Seven kinds of polydextroses with molecular weights ranging from 10 to 500 kDa were used as standards. The molecular weight of AX in the sample was analyzed by comparing the retention times of the molecular-weight standard curve of the standards [16,35].

The contents of arabinose and xylose in AX and its degree of branching were determined by the flame ionization detector (FID) in Aligent 7890a gas chromatography device (GC, Aligent, Santa Clara, CA, USA) equipped with DB-5 capillary column (30 m × 0.25 mm × 0.25 μm) [36]. The chromatographic column was set as follows: 50–230 °C at 10 °C/min.

### 2.3. Preparation of the AX/WS Complexes

A quantity of 1.00 g of WS was weighed and mixed evenly with 0.0005 g, 0.005 g, 0.01 g, and 0.02 g (*w*/*w*) of AX. A quantity of 15 mL of distilled water was added, and it was heated in a shaking water bath at 95 °C for 30 min, and 0%, 0.05%, 0.5%, 1%, and 2% AX/WS composite solutions were obtained respectively. The AX/WS gel was cooled to room temperature (25 °C), placed in a −80 °C refrigerator, and freeze-dried for subsequent analysis.

### 2.4. Solubility and Swelling Capacity

The samples prepared in Section 2.3 were cooled to room temperature and centrifuged (5000 rpm, 15 min), then the precipitate was weighed, the supernatant was dried to constant weight at 105 °C, and it was weighed as W_s_ to calculate the starch solubility index *SI* (%); the wet weight of the precipitate was weighed as *W_r_* to calculate the starch swelling power *SP* (g/g).

(1) $$\mathrm{SI}\;(\%)=\frac{W_{S}}{W_{i}}\text{×}100\;$$

(2) $$SP\;\left(g/g\right)=\frac{W_{r}}{W_{i}}\times \left(100-SI\right)$$

### 2.5. Determination of Leakage Amylose Content and Water Holding Capacity

A quantity of 1 mL of the supernatant was mixed with 6 mL of NaOH (0.33 M), heated in a water bath at 95 °C for 30 min, cooled, and then centrifuged for 10 min (4800 rpm). A quantity of 0.1 mL of the supernatant was taken and mixed with 5 mL of trichloroacetic acid (0.5%, *v*/*v*), the pH of this solution was controlled between 5.0–6.0, and then 0.01 N iodine–potassium iodide indicator was added and mixed well and reacted at room temperature for 30 min. The absorbance was measured at 620 nm and the amount of amylose dissolution was calculated using amylose as the standard.

A quantity of 20.00 g of the gelatinized sample was accurately weighed and placed into a 50 mL centrifuge tube. After standing at room temperature for 1 h, it was centrifuged at a speed of 8000 rpm for 10 min and the weight of the supernatant was accurately weighed. The following formula was used to calculate the water separation rate of the sample:

(3) $$Water\;separation\;rate\;\left(\%\right)=\frac{Weight\;of\;supernatant}{total\;weight}\times 100\;$$

### 2.6. Rheological Property Measurement

The completely gelatinized composite samples were cooled to room temperature. The rheological properties of the composite fresh gel were measured using the MARS 60 rheometer (Thermo, Waltham, MA, USA). First, the starch gel sample was placed on a 20 mm plate and balanced for 2 min. Then the test temperature was set to 25 °C and the Gap value to 500 μm. A steady-state rate scan was conducted on the starch gel sample within the shear rate range of 0.01–1000 s^−1^; finally, the Power-law model was used to perform regression fitting on the measured data points:

(4) $$ɳ=K\gamma^{n-1}$$

ɳ represents the apparent viscosity, (Pa·s); K represents the consistency coefficient, (Pa·sn); γ represents the shear rate, (s^−1^); n represents the fluid characteristic index.

The test temperature was set to 25 °C and the strain to 1%, and the dynamic rheological properties of the starch gel sample were measured within the frequency range of 0.1–10 Hz. The storage modulus (G′), loss modulus (G″), and loss tangent value (tanδ = G″/G′) of the sample were obtained through the system software of the instrument.

### 2.7. X-ray Diffraction

The X-ray diffraction patterns of the samples were obtained in the range of 3° to 40° (2θ) using a D/teX Ultra X-ray diffractometer (Ultima IV, Rigaku, Japan) under conditions of 40 kV and 40 mA with a scanning speed of 5°/min and a step size of 0.02°.

### 2.8. Fourier-Transform Infrared Spectroscopy (FTIR) Measurement

The FTIR spectra were measured by a Fourier-transform infrared spectrometer (VERTEX 80V, Bruker, Germany). A total of 1.5 mg of the sample and 150 mg of potassium bromide powder were accurately weighed, ground thoroughly, pressed into tablets, and then vacuumized. Each sample tablet was placed on the sample holder for scanning, the resolution was set to 4 cm^−1^, and 32 scans were made within the scanning band of 400–4000 cm^−1^.

### 2.9. Environmental Scanning Electron Microscope (SEM)

The freeze-dried samples were taken and stuck on the carrier stage with double-sided conductive adhesive. The microstructures of the WS-AX composite gel samples were observed using a SEM (Zeiss EVO MA10, Oberkochen, Germany). All samples were observed and photographed at a magnification of 400 times, with an acceleration voltage of 15 kV.

### 2.10. In Vitro Digestion

Englyst’s method [37] was used to simulate the in vitro digestion analysis of starch products. A quantity of 150 mg of flour products was weighed in a centrifuge tube, 5 mL of 5 g/L pepsin solution was added, and it was incubated in a water bath at 37 °C for 30 min. A quantity of 5 mL of 0.25 M sodium acetate buffer solution (pH = 5.2) was added to the solution after water bath, then 2.5 mL of the mixed solution of pancreatic enzymes and amyloglucosidase was added. The flour products were hydrolyzed by shaking at 37 °C for 4 h (samples were taken every 20 min in the first 2 h and every 1 h in the last 2 h). Immediately after sampling, the hydrolysis reaction was terminated with absolute ethanol. After centrifugation, the glucose content in the hydrolyzate was measured using a D-glucose detection kit. *G*_20_ and *G*_120_ represented the glucose contents at 20 and 120 min of hydrolysis. Starch components could be divided into three parts: rapidly digestible starch (*RDS*), slowly digestible starch (*SDS*), and resistant starch (*RS*), which could be calculated by the following formulas:

(5) $$RDS\left(\%\right)=G_{20}\times 0.9÷TS\times 100$$

(6) $$SDS\left(\%\right)=\left(G_{120}-G_{20}\right)\times 0.9÷TS\times 100$$

(7) $$RS\left(\%\right)=\left(TS-\left(RDS+SDS\right)\times 100\right)\times 0.9÷TS$$

In Formulas (5)–(7), *G*_20_ and *G*_120_ represent the glucose content at 20 and 120 min of hydrolysis and TS represents the total starch content of the sample.

### 2.11. Predicted Glycemic Index (pGI)

During the in vitro digestion simulation of starch, the mass (mg) of glucose in the hydrolyzate at different time periods was calculated. Through the first-order kinetic equation, Equation (8), it was fitted to obtain the digestion kinetic characteristic parameters and the pGI value. Among them, the hydrolysis degree (HI) was expressed as the ratio of the area under the hydrolysis curve of the composite system to the area under the hydrolysis curve of the control group. The pGI of the reference substance was defined as 100 and the pGI of the composite system sample was calculated through the equation.

(8) $$C=C_{\infty }\left(1-e^{-kt}\right)$$

(9) $$CAUC=C_{\infty }\left(t-t_{0}\right)-\frac{C_{\infty }}{k}\left[1-{exp}^{-k\left(t-t_{0}\right)}\right]$$

(10) $$HI\left(\%\right)=\frac{{AUC}_{S}}{{AUC}_{C}}\times 100$$

(11) pGI=39.71+0.549HI

Here, *HI* is the glucose hydrolysis rate at the final hydrolysis equilibrium (%); *k* is the kinetic constant (min^−1^); *t* is the hydrolysis time (min).

### 2.12. Statistical Analyses

All experiments were repeated three times. Each set of data was expressed as a mean ± standard deviation, and the data were processed using IBM SPSS Statistics version 21.0 (IBM, Armonk, NY, USA) for significance analysis, where *p* < 0.05. All images were plotted using Origin 2019 and Adobe Illustrator 2019, version 23.0.1, software.

## 3. Results and Discussions

### 3.1. Compositional Components of the Cell Wall of Wheat Bran

The WEAX in wheat bran mainly originates from the cell wall of the aleurone layer [38,39]. As shown in Table 2, the content of TAX in the eight kinds of wheat accounted for 11.90–15.79% of their dry weights and the content of WEAX accounted for 0.51–0.89% of their dry weights. The content of TAX increased with increases in altitude in the wheat-growing areas; the highest content was found in wheat-LWW. The contents of WEAX in the wheat bran from eight different production areas differed significantly (*p* < 0.05). The altitude has a significant influence on temperature changes; the low temperature during the grain filling period of wheat is beneficial for increasing the thickness of the aleurone layer, thus increasing the content of AX in wheat bran. The degrees of branching (Ara/Xyl) of AXs were 0.50–0.55. Based on the above experimental results, the wheat bran of wheat-LWW was used to extract AX as a source of high-quality dietary fiber.

### 3.2. Gel Properties

The solubility and swelling degree of starch gel after the addition of AX are shown in Table 3. It can be seen from Table 3 that the solubility of WS increased after the addition of AX (*p* < 0.05). With an increase in the addition ratio, the solubility decreased from 20.35% to 15.13%, the swelling capacity decreased from 15.62 (g/g) to 12.17 (g/g), and the leakage amylose significantly decreased from 30.62% to 24.24% (*p* < 0.05). Previous studies have shown that the addition of AX will affect the intermolecular interaction of starch molecules in the crystalline or amorphous domains [40] and also affect the degree of interaction between starch and water molecules [41]. In addition, the added AX will also affect the swelling capacity, solubility, leakage starch content, and water holding capacity of starch [40]. Therefore, the increased solubility and decreased swelling capacity of the composite system may be such that the addition of AX hinders the rapid entry of water molecules into starch granules, thereby preventing the water absorption and swelling of starch during heating to a certain extent.

### 3.3. Rheological Analysis

**Figure 1:**
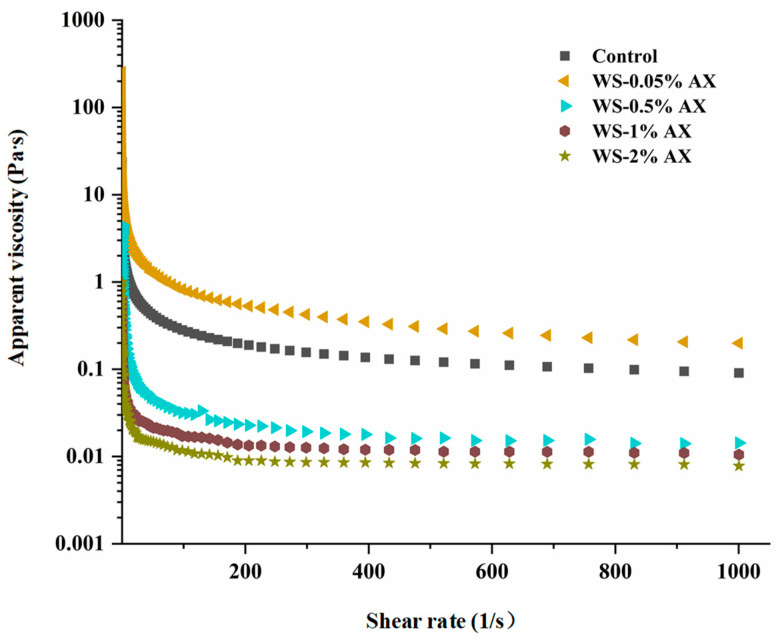
Variation in apparent viscosity of wheat starch in the presence of different concentrations of AX. Note: AX: Arabinoxylan; WS: wheat starch. The values are means ± SDs.

The steady-state shear rheological properties of starch gel with the addition of different concentrations of AX are shown in Figure 1. At a shear rate of 1–1000 s^−1^, all samples exhibited shear-thinning pseudoplastic phenomena, that is, the apparent viscosity of all samples decreased as the shear rate increased, which also indicated that the AX/WS gel was a typical non-Newtonian fluid. It can be observed from the figure that compared with the blank group, when the concentration of AX added was 0.05%, the apparent viscosity of the starch gel increased. However, as the concentration gradually increased, the apparent viscosity of the AX/WS gel system decreased and was lower than that of the blank group. Therefore, the results showed that when a low concentration of AX was added, the entanglement between AX and WS molecules increased the apparent viscosity of the gel system, showing a stronger shear-thinning effect, while when a high concentration of AX was added, the pseudoplasticity of the starch gel system gradually weakened and the shear resistance weakened, but its fluidity improved [16,31,40].

**Figure 2:**
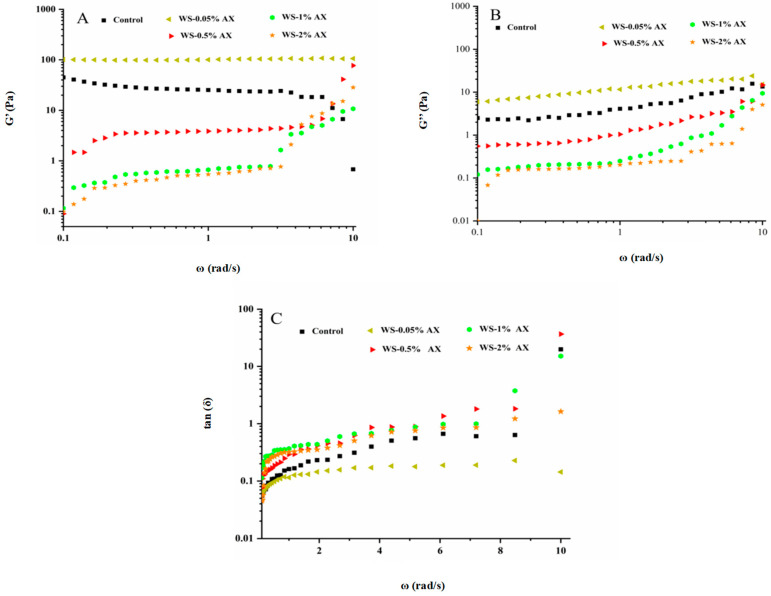
Variation in (**A**) energy storage mode (G′), (**B**) loss mode (G″), and (**C**) loss angle tangent (tan δ) of wheat starch in the presence of different concentrations of AX. Note: AX: Arabinoxylan; WS: wheat starch. The values are means ± SDs.

The viscoelasticity of starch gel can also determine the texture of starchy foods [41]. Dynamic rheology judges whether a sample is dominated by viscosity or elasticity through the viscoelastic modulus of the sample [17]. G′ and G″ respectively reflect the storage modulus and loss modulus of dynamic deformation. The former refers to reversible deformation and the latter refers to irreversible deformation while the loss tangent value (tan δ) is the ratio of G″ to G′ [32]. According to Figure 2, with an increase in AX concentration, the dynamic viscoelasticity of WS paste changed. In addition, it can also be observed from Figure 2 that the G′ of all gel samples was significantly higher than the G″, and tan α < 1, which indicates that all samples exhibited typical viscoelasticity and solid-like behavior. Compared with the blank group, when the AX concentration was 0.05%, the G′ and G″ of the AX/WS gel system increased. This might have been because the low concentration of AX could fully interact with WS, strengthening the network structure of the AX/WS gel system, but with an increase in AX addition, it could be found that the G′ and G″ of the AX/WS gel system gradually decreased. Among them, the G′ and G″ of the AX/WS gel system with 2% AX added were the lowest. This might have been because after some AX interacted with WS, the remaining AX wrapped on the surfaces of WS particles, hindering the water absorption and swelling of starch particles, reducing the leaching of leakage amylose, making it difficult for the AX/WS gel system to form an ordered conformation, thereby weakening the gel network structure.

Tan δ is the ratio of G″ to G′, and the larger the tan δ value is, the greater the proportion of viscosity in the system and the stronger the fluidity is; conversely, the proportion of elasticity is larger. It can be found from Figure 2C that when the AX addition concentration was 0.05%, the tan δ value of the AX/WS gel system was lower than that of the blank group, indicating that an AX concentration of 0.05% could reduce the fluidity of the AX/WS gel system and had the property of an elastic fluid bias; when 0.50–2.00% AX was added, the tan δ value of the AX/WS gel system was higher than that of the blank group, indicating that an AX concentration of 0.50–2.00% could enhance the fluidity of the AX/WS gel system, presenting a strong viscosity property and resulting in weakened elasticity in the system.

### 3.4. X-ray Diffraction Analysis

**Figure 3:**
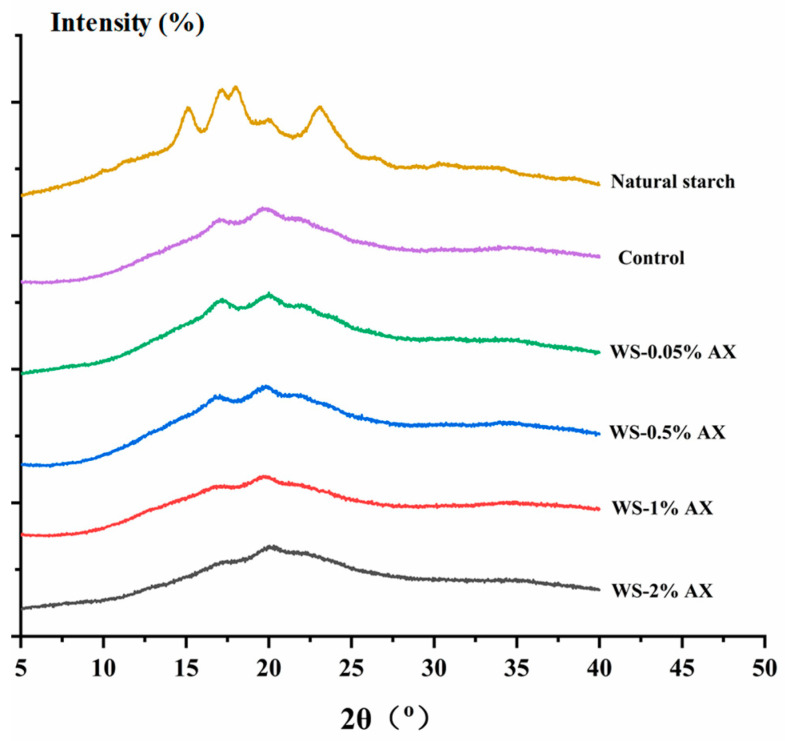
X-ray diffractograms of wheat starch gels with different concentrations of AX added. Note: AX: Arabinoxylan; WS: wheat starch. The values are means ± SDs.

As shown in Figure 3, natural starch has a typical A-type structure, with strong diffraction peaks at 15° and 23°, a weak diffraction peak at 20°, and characteristic double peaks at 17° and 18°. However, after starch gelatinization to form a gel, the A-type crystalline structure and most of the crystalline peaks disappear, and there are only weak diffraction peaks at 17° and 20°, and the diffraction peaks are relatively wide, indicating that the crystal structure of natural WS has been destroyed, and the diffraction pattern changes from type A to type B. This is due to the gradual densification of the structure of amylose and amylopectin during the starch gelatinization and retrogradation process. The diffraction peak at 17° is mainly caused by the continuous retrogradation of amylopectin during storage. Starch retrogradation involves not only a change in amylopectin in the starch system but also relates to some amylose. The diffraction peak at 20° is produced by the complex formed by amylose and fatty acids and phospholipids, showing a V-type characteristic. This actually indicates that the addition of AX does not affect the crystal type of starch. It can be seen from Figure 3 that as the concentration of AX increases, the intensity of the diffraction peak gradually decreases, indicating that the addition of AX can inhibit the recrystallization of amylopectin to a certain extent, and the inhibitory effect of adding 2.00% AX was better in this study. Yan et al. (2021) reported a similar phenomenon [42].

### 3.5. Fourier-Transform Infrared Spectroscopy Analysis

**Figure 4:**
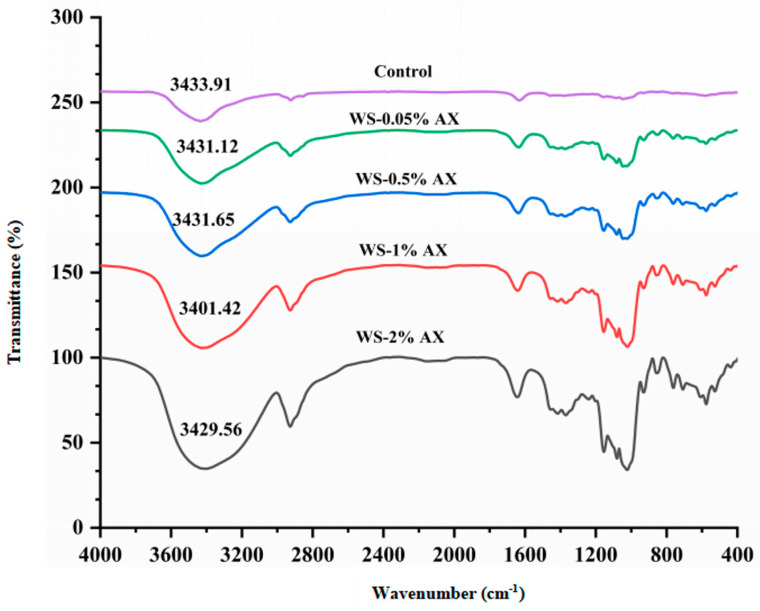
FTIR results for wheat starch gel with different concentrations of AX. Note: AX: Arabinoxylan; WS: wheat starch. The values are means ± SDs.

The infrared spectra (400–4000 cm^−1^) of starch gel with different concentrations of AX added are shown in Figure 4. It can be found from the figure that after adding different concentrations of AX, the infrared spectrum images of the AX/WS gel system did not change significantly, indicating that there was no covalent bond with a strong interaction force generated between AX and WS, but there was a combination through weak forces. Fourier infrared can be used to deeply understand the short-range ordered structures of starch molecules. The strong and wide peak of starch in the wavelength range of 3000–3600 cm^−1^ was related to the stretching vibration of O-H, the absorption peak in the wavelength range of 2800–3000 cm^−1^ was produced by the stretching vibration of C-H, the peak at 1000–1200 cm^−1^ was related to the C-O-C bond, and the wavelength range of 800–1200 cm^−1^ was in the fingerprint region of starch. In addition, the absorption peak representing the bending vibration of water molecules would appear near 1646 cm^−1^. A wide single peak was observed at about 3400 cm^−1^, indicating the presence of hydrogen bonds within the molecule, and with an increase in AX concentration, the absorption peak became wider, indicating that an increase in AX may have interfered with the O-H stretching vibration. Moreover, compared with the blank group, when different concentrations of AX were added, the peaks at 2800–3000 cm^−1^ and 1000–1200 cm^−1^ decreased, indicating that C-H and C-O-C were partially destroyed.

### 3.6. Electron Microscope Analysis

**Figure 5:**
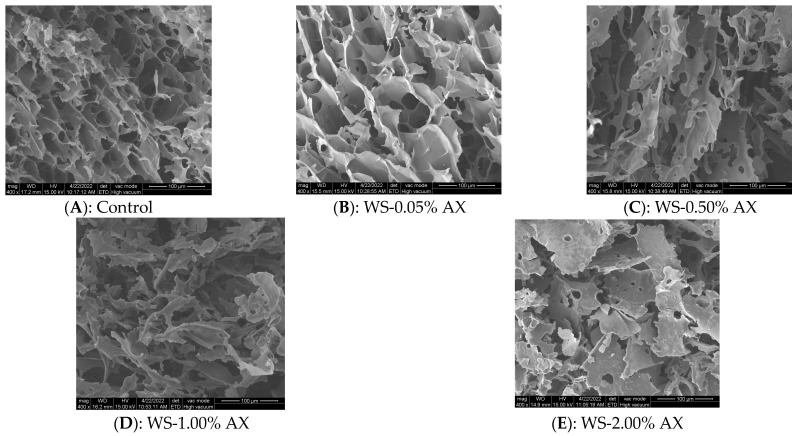
Effects of adding different concentrations of AX on the microstructure of wheat starch gels. Note: (**A**): Control; (**B**): WS-0.05% AX; (**C**): WS-0.50% AX; (**D**): WS-1.00% AX; (**E**): WS-2.00% AX. AX: Arabinoxylan; WS: wheat starch. The values are means ± SDs.

SEM images of WS gel with different concentrations of AX added are shown in Figure 5. Due to the expansion of particles and the leakage of starch chains during the heat treatment process, the re-aggregation and arrangement of WS molecules and the evaporation of water during the freeze-drying process of starch gel resulted in a porous network structure for all gels. It can be observed from Figure 5A that the SEM image of the WS gel presented an irregular and loose porous sheet structure, which was consistent with the experimental results of Xie et al. [16]. In the lyophilized WS-0.05% AX complex, the pores of the gel system were larger. However, as the addition amount of AX increased, the gel network structure gradually weakened and were replaced by a brittle sheet structure, with almost no gel network structure formed, which may have been because the addition of AX inhibited the expansion and gelatinization of some WS particles.

### 3.7. In Vitro Digestion Analysis

The interaction between starch and dietary fiber can inhibit the in vivo digestion of starch [43], thereby reducing the postprandial blood glucose response [44]. As shown in Table 4, compared with the starch gel without AX added, the total hydrolysis rate of WS significantly decreased (*p* < 0.05) after the addition of AX. Compared with the blank, the addition of AX led to a decrease in the RDS, and it decreased with an increase in the addition amount. Compared with the blank, the addition of AX led to an increase in the RS, and it increased with an increase in the addition amount. Among all bread samples, the hydrolysis rate of 2.00% AX was the lowest, of which the contents of RDS and SDS were 40.02% and 36.61%, and RS was as high as 25.31%. The in vitro kinetic model was used to fit to predict the glycemic index, and the calculated hydrolysis rate results are shown in Table 4. The pGI values of the complexes after the addition of AX were lower than those of the blank samples. The pGI was between 90.00% and 93.27%. Among the available values, the pGI of 2.00% AX was 90.00%, indicating that the AX extracted in this study reduced the starch hydrolysis rate [45]. The hydrolysis index can be used to predict the glycemic index. A low hydrolysis index indicates a lower predicted glycemic index value. This indicates that within a certain concentration range, AX can inhibit the degree of starch hydrolysis and reduce the postprandial glycemic index. Therefore, the addition of AX to starch-based foods is helpful for controlling postprandial blood sugar and insulin levels and has broad prospects for development in the application of hypoglycemic foods in the future [46,47].

## 4. Conclusions

The AX contents in wheat bran from regions with different longitudes, latitudes, and altitudes were significantly different. Among the eight types of wheat, the content of TO-AX accounted for 11.90–15.79% of the dry weight of wheat bran. The content of AX in wheat bran rose along with an increase in the altitude of the wheat production area. The content of WE-AX in wheat-LWW from the high-altitude production area was as high as 0.85%. After adding the AX values of different concentrations ranging from 0.05% to 2% to the WS system, the WS-AX gel network structure was changed. When a high concentration of AX was added, the pseudoplasticity of the starch gel system gradually weakened, and the shear resistance decreased, G′ and G″ gradually decreased, enhancing the fluidity of the AX/WS gel system. The hydrolysis rate of bread starch with 2% AX added was the lowest, of which the contents of RDS and SDS were 40.02% and 36.61%, respectively, and the RS was as high as 25.31%. In the future, the geographical variations in the AX structures of wheat cell walls in different wheat production areas need to be further studied, and the influence on the digestive properties of starch should also be further investigated.

## Figures and Tables

**Table 1:** Basic information on wheat samples.

Serial Number	Wheat Production Area	Abbreviation	Variety	Latitude	Longitude	Altitude (m)	T_1_ (°C)	T_2_ (°C)
1	Heze, Shandong	JHZ	Jimai 22	N35°14′	E115°29′	53	26	12
2	Bengbu, Anhui	HBB	Huaimai 22	N32°55′	E117°24′	23	23	13
3	Shangqiu, Henan	ZSQ	Zhengmai 379	N34°25′	E115°40′	45	23	13
4	Suqian, Jiangsu	BSQ	Bainong 307	N32°15′	E117°69′	15	22	12
5	Luoyang, Henan	ZLY	Zhengmai 136	N34°40′	E112°20′	147	24	13
6	Baoji, Shaanxi	XBJ	Xinong 226	N34°22′	E107°14′	890	27	15
7	Yuncheng, Shanxi	JYC	Jindong 19	N35°06′	E111°06′	1205	23	11
8	Wuwei, Gansu	LWW	Longchun 44	N37°56′	E102°38′	1720	25	6

Note: T_1_ is daily average high temperature during grain filling period; T_2_ is daily average low temperature during grain filling period.

**Table 2:** Wheat bran components.

Serial Number	Abbreviation	Yield (%)	Purity (%)	WEAX Content (%)	TAX Content (%)	Substitution Rate (Ara/Xyl)	Molecular Weight Mw (kDa)
1	JHZ	9.53	82.91	0.57 ± 0.03	11.90 ± 0.15	0.51	111.32
2	HBB	9.12	82.19	0.59 ± 0.05	12.32 ± 0.28	0.51	109.47
3	ZSQ	10.29	81.35	0.63 ± 0.03	12.52 ± 0.30	0.53	113.33
4	BSQ	10.07	83.77	0.51 ± 0.03	12.02 ± 0.29	0.51	117.32
5	ZLY	10.36	80.12	0.55 ± 0.04	12.42 ± 0.26	0.50	115.39
6	XBJ	10.98	83.67	0.64 ± 0.06	12.88 ± 0.31	0.51	113.15
7	JYC	10.99	82.72	0.65 ± 0.03	12.89 ± 0.22	0.55	120.05
8	LWW	12.72	83.55	0.89 ± 0.05	15.79 ± 0.25	0.52	121.95

Note: WE-AX and TO-AX are water-soluble Arabinoxylan and total Arabinoxylan, respectively. Each set of data is represented by the average of three groups ± standard deviation.

**Table 3:** Gel properties of the complexes.

Sample	Solubility (%)	Swelling Capacity (g/g)	Leaked Amylose Content (%)	Syneresis Rate (%)
WS	20.35 ± 0.14	15.62 ± 0.12	30.62 ± 0.15	5.19 ± 0.06
WS-0.05% AX	17.23 ± 0.16	13.79 ± 0.13	27.32 ± 0.16	12.13 ± 0.05
WS-0.50% AX	16.57 ± 013	13.37 ± 0.14	26.66 ± 0.13	16.32 ± 0.07
WS-1.00% AX	16.17 ± 0.15	12.78 ± 0.18	26.55 ± 0.12	21.61 ± 0.05
WS-2.00% AX	15.13 ± 0.12	12.17 ± 0.18	24.24 ± 0.13	25.22 ± 0.09

Note: AX: Arabinoxylan; WS: wheat starch. The values are means ± SDs.

**Table 4:** Digestibility model parameters of samples.

Sample	*RDS* (%)	*SDS* (%)	*RS* (%)	*HI*	pGI
C	49.22 ± 0.32	34.93 ± 0.33	17.85 ± 0.30	100.00 ± 0.00	94.61 ± 0.00
WS-0.05% AX	46.83 ± 0.31	33.93 ± 0.35	19.24 ± 0.32	97.56 ± 0.45	93.27 ± 0.52
WS-0.5%AX	43.91 ± 0.18	35.53 ± 0.30	20.56 ± 0.29	95.32 ± 0.44	92.04 ± 0.36
WS-1%AX	42.15 ± 0.22	35.07 ± 0.26	22.78 ± 0.26	93.78 ± 0.41	91.20 ± 0.33
WS-2%AX	40.02 ± 0.19	36.61 ± 0.23	25.31 ± 0.29	91.61 ± 0.42	90.00 ± 0.32

Note: Each set of data is expressed by the average of three groups ± standard deviation. RDS: rapidly digestible starch, SDS: slowly digestible starch; RS: resistant starch; HI: hydrolysis indices; pGI: predicted glycemic indices.

## Data Availability

The data presented in this study are available on request from the corresponding author due to privacy.
